# Association of Genetic Polymorphisms in *CDH1* and *CTNNB1* with Breast Cancer Susceptibility and Patients' Prognosis among Chinese Han Women

**DOI:** 10.1371/journal.pone.0135865

**Published:** 2015-08-18

**Authors:** Yu-Mian Jia, Yun-Tao Xie, Ya-Jun Wang, Ji-Yuan Han, Xin-Xia Tian, Wei-Gang Fang

**Affiliations:** 1 Department of Pathology, Key Laboratory of Carcinogenesis and Translational Research (Ministry of Education), School of Basic Medical Sciences, Peking University Health Science Center, Beijing, China; 2 Department of Pathology, Peking University Third Hospital, Beijing, China; 3 Breast Center, Peking University School of Oncology, Beijing Cancer Hospital & Institute, Beijing, China; Centro di Riferimento Oncologico, IRCCS National Cancer Institute, ITALY

## Abstract

This study aims to investigate whether the germline variants in *CDH1* and *CTNNB1* would affect breast cancer susceptibility and patients’ prognosis among Chinese Han women using a haplotype-based association analysis. We genotyped 12 haplotype-tagging single nucleotide polymorphisms (htSNPs) in *CDH1* and *CTNNB1* among 1,160 BC cases and 1,336 age-matched cancer-free controls using the TaqMan^®^ Genotyping Assay. For association analyses of germline variants with breast cancer susceptibility, the results showed that rs7200690, rs7198799, rs17715799, rs13689 and diplotype CGC/TGC (rs7200690 + rs12185157 + rs7198799) in *CDH1* as well as rs2293303 in *CTNNB1* were associated with increased breast cancer risk. In addition, the Generalized Multifactor Dimensionality Reduction (GMDR) and logistic regression analysis predicted an interaction on breast cancer risk between rs17715799 and rs13689 as well as rs13689 and menarche-FFTP (First Full-Term Pregnancy) interval. For survival analyses, the results demonstrated that the minor allele homozygotes of rs13689 and haplotype TGC in *CDH1* were linked with unfavorable event-free survival of breast cancer, whereas, rs4783689 of *CDH1* showed the opposite effect under dominant model. Notably, the stratified analysis revealed that rs7186053 was associated with favorable event-free survival among patients with estrogen receptor (ER)-positive, progesterone receptor (PR)-positive or lymph node metastasis negative patients. Moreover, rs7200690 and rs7198799 in *CDH1* as well as rs4533622 in *CTNNB1* were associated with worse event-free survival among patients with clinical stage 0-I tumors. This study indicated that the genetic polymorphisms of *CDH1* and *CTNNB1* were associated with breast cancer susceptibility and patients’ prognosis.

## Introduction

Breast cancer (BC) is, by far, the most frequent cancer and the most likely common cause of cancer death among women [1]. Epithelial-mesenchymal transition (EMT) has been regarded as a potentially important event in the metastatic spread of tumor cells, in which epithelial tumor cells acquire a more motile and invasive phenotype and escape from the primary tumor [2, 3]. In addition, induction of EMT also elicits numerous other properties that likely contribute to tumor development and progression including carcinogenesis, stem cell-like generation, resistance to chemotherapy and senescence, and evasion of the immune system [3, 4]. The *CDH1* and *CTNNB1* genes, which encode the proteins E-cadherin and β-catenin respectively, are two crucial factors involved in the regulation of the EMT process [5], therefore, we proposed the hypothesis that single nucleotide polymorphism (SNP) in *CDH1* and *CTNNB1* genes would contribute to BC development and progression.

E-cadherin, as a tumor- and an invasion-suppressor [6], is a homophilic cell-to-cell adhesion protein localized to the adherens junctions of all epithelial cells [7]. In breast cancer, partial or total loss of E-cadherin expression correlates with loss of differentiation characteristics, acquisition of invasiveness, increased tumor grade, metastatic behavior and poor prognosis [8]. Somatic inactivation of the *CDH1* gene by mutations or allelic deletions, as well as promoter methylation, is frequent in BC [9]. Although the somatic and germline mutations in *CDH1* is restricted to lobular breast tumors [8–11], ductal breast carcinomas often show strikingly reduced E-cadherin mRNA and protein expression [8]. This reduced expression could be explained by some mechanisms such as chromatin rearrangements, hypermethylation and alterations in trans-factor binding [8]. SNP, a common type of genetic variation, also contribute to this reduced expression. A functional polymorphism (rs16260, −160 C/A) in promoter of *CDH1* was found to reduce E-cadherin expression [12], and linked with 30% increased risk of BC by the minor allele A [13]. In addition, several other SNPs in *CDH1* such as rs13689, rs2059254 and rs12919719 were found to be associated with BC susceptibility [14].

β-catenin has two roles in the cells. It forms a functional cadherin-catenin adhesive complex and involves in cell-cell adhesion in the membrane, while its nuclear pool participates in signaling pathways and regulates a remarkable variety of cellular process such as cell proliferation, cell survival and migration [15]. β-catenin involves in the carcinogenesis of infiltrative ductal carcinoma [16], and is associated with increased BC risk and worse prognostic phenotype [16–18]. Although somatic mutation of *CTNNB1* is rare in BC [19, 20], mounting evidences have revealed that the somatic mutations in *CTNNB1* are often associated with the upregulation of β-catenin and the pathogenesis of endometrioid-type of endometrial cancer and ovarian cancer [21, 22]. Germline mutation in *CTNNB1* is not found in BC. It is reported that null mutations of β-catenin in mice models result in gastrulation defects and embryonic lethality [23]. However, several germline variants of *CTNNB1* were found to be associated with BC risk [24, 25]. One study found that rs4135385 was linked with increased BC risk [24], while another study indicated that rs4135385 was associated with decreased BC risk [25].

Until now, there have been no comprehensive association studies of germline variants of the two genes with BC among Chinese Han population. Based on linkage disequilibrium (LD), a set of associated SNP alleles in a region of a chromosome forms a “haplotype”, while a pair of haplotypes forms a diplotype. It is believed that applying a minority of informative SNPs called haplotype-tagging SNPs (htSNPs) can capture the contribution of almost all of the SNPs on a target gene to a specific phenotype [26, 27]. In this study, we selected htSNPs in these two genes and comprehensively investigated the associations of genetic polymorphisms of *CDH1* and *CTNNB1* with BC susceptibility and event-free survival in Chinese Han population.

## Materials and Methods

### Study population

This case-control study included 1,160 female BC patients and 1,336 cancer-free controls. All the 1,160 cases were pathologically diagnosed with primary infiltrating ductal carcinoma of the breast at the Beijing Cancer Hospital in China during the period 1995–2007. Their epidemiological information was obtained from their clinical records, including age at diagnosis, height, weight, age at menarche and/or menopause, menopause status, age at first full-term pregnancy and family history of cancer in first-degree relatives. All the clinicopathological parameters were also collected from their clinical records, these including estrogen receptor (ER), progesterone receptor (PR), human epidermal growth factor receptor 2 (Her2), tumor size, lymph node status, clinical stage (based on the 6th edition of TNM staging of the American Joint Committee on Cancer system), chemotherapy and endocrine therapy status [28]. The event-free survival time was defined as the time from the surgery to the breast events such as breast carcinoma recurrence, metastasis, and death caused by BC. Cases were censored if the patients were still alive or voluntarily withdrew or died of a cause other than BC before the latest follow-up (August 31, 2010) [29]. The median follow-up time after surgery was 3.4 years. Of the 1,160 cases, 51 cases had no operation, 17 cases lost to follow-up and 1 case died of unknown cause. Thus, there remained 1091 cases in the event-free survival analysis. The 1,336 controls were selected from cancer-free women participating in a community-based screening programme for non-infectious diseases conducted in Beijing, China. The selection criteria included no history of cancer, Chinese Han ethnic background and age-matched to cases (same 5-year group) [29]. All eligible controls completed an epidemiological questionnaire.

### SNPs Selection

All haplotype-tagging SNPs (htSNPs) in *CDH1* and *CTNNB1* were selected according to the methods described previously [27]. We identified 10 htSNPs in the *CDH1* locus [spanning from 2 kb upstream to 2 kb downstream of *CDH1* gene; minor allele frequency (MAF) > 5% in Han Chinese in Beijing population (CHB)], these including 4 SNPs in intron 2 [rs7200690 (C>T), rs12185157 (A>G), rs7198799 (C>T), rs17715799 (A>T)], 3 SNPs in intron 3 [rs2011779 (T>C), rs10431923 (T>G), rs7186053 (G>A)], rs6499199 (C>T) in intron 10, rs4783689 (C>T) in intron 11 and rs13689 (T>C) in 3’UTR. We identified 3 htSNPs in the *CTNNB1* locus [spanning from 2kb upstream to 2kb downstream of *CTNNB1* gene; MAF > 5% in CHB], these being: rs4533622 (C>A) in intron 2, rs4135385 (G>A) in intron 14 and rs2293303 (C>T) in exon 17 (synonymous). Due to the failure of rs2011779 in genotyping, therefore, only the remaining 12 SNPs were included in the following study.

### DNA isolation, genotyping assay, and quality control

Genomic DNA was extracted from blood leukocytes. Genotyping was carried out by the ABI 7900HT Real-Time PCR System (Applied Biosystems, Foster City, CA, USA) using the TaqMan^®^ Assay according to the manufacturer’s instructions (Applied Biosystems, Foster City, CA, USA). Primers and probes were supplied directly by Applied Biosystems as Assays-by-Design and Assays-on-Demand products, and PCR conditions were the same as described previously [30]. Positive and negative controls were contained in each 384 genotyping plate. As a quality control, genotyping in 2% of the samples were repeated, and the concordance between the duplicates was more than 99%.

### LD block determination and haplotype construction

Linkage Disequilibrium (LD) plots of Lewontin coefficient (D’) and squared correlation coefficient (r^2^) between the genotyped SNPs were produced based on our genotyping data using the Haploview program [31]. Then haplotype blocks in cases and controls were respectively reconstructed with the Haploview 4.2 software. For haplotype estimation, the most probable haplotypes for each participant were estimated using the SAS 9.1 PROC HAPLOTYPE procedure [28].

### Statistical analysis

Differences in demographic characteristics and selected variables between cases and controls were compared by two-sided chisquare (x^2^) test or student’s t test. Hardy-Weinberg equilibrium was evaluated for each SNP using a one-degree of freedom goodness-of-fit test among the controls, and the cut-off threshold we used was 0.05 [27]. A two-sided x^2^ test was employed to compare differences in the distributions of genotypes and alleles between cases and controls. Each genotype was assessed according to codominant, dominant and recessive models. In addition, Cochran-Armitage trend test was performed to estimate the association between BC risk and allele dose in each SNP (P trend). Furthermore, Odds ratios (ORs) with 95% confidence intervals (95% CIs) were calculated to evaluate the effects of genotypes or haplotypes on BC risk using both univarirate and multivariate unconditional logistic regression models, adjusted for age at menarche, age of first birth and family history of cancer in first-degree relatives [29, 30].

Generalized Multifactor Dimensionality Reduction (GMDR) method (GMDR Beta program, version 7 software) combining with logistic regression models were used to analyze gene-gene and gene-environment interactions. GMDR is a nonparametric and genetic model-free alternative to linear or logistic regression for detecting and characterizing nonlinear interactions among discrete genetic and environmental attributes. The GMDR method, compared with the MDR method, can use score statistics to process both quantitative and dichotomous traits and permits adjustment for covariates. Gene-gene interactions were examined for one- to five-locus models by GMDR, which included 12 SNP loci and adjusted for age, Body Mass Index (BMI), age at menarche, age of first birth, and family history of cancer in first-degree relatives. Gene-environment interactions were also examined for one- to five-order models by GMDR, which handled 16 attributes including 12 SNP loci, age, BMI, family history of cancer in first-degree relatives and menarche-FFTP (First Full-Term Pregnancy) interval [27] and adjusted for 5 covariates including age, BMI, age at menarche, age of first birth, and family history of cancer in first-degree relatives. We further explored the effects of these interactions on BC risk using logistic regression models.

Survival estimates were computed using the Kaplan-Meier method, and differences between survival times were evaluated using the log-rank test. To further investigate the associations of clinicopathological parameters, genotypes and haplotypes with event-free survival, hazard ratio (HR) and 95% CIs were calculated using univariate and multivariate Cox proportional hazards model, adjusted for ER status, PR status, Her2 status, tumor size, clinical stage, lymphnode metastasis, chemotherapy and endocrine therapy status. Stratified association analysis was conducted to determine the effects of interaction between genetic variants and clinical risk factors on BC survival. All statistic analyses were done with Statistic Analysis System software (v.9.1; SAS Institute, Cary, NC). A two-sided P value <0.05 was considered statistically significant.

### Ethic statement

Written informed consents were obtained from all control samples. Breast cancer samples were collected initially for research purposes in the tissue/blood biobank. Written consents were obtained from the BC patients who can read and write. For the BC patients who cannot read and write, verbal consent was obtained and written consent was signed by her next of kin. The written consent procedure was approved by IRB. The data/samples were used anonymously. PKU IRB approved our application to waive informed re-consent for the already collected BC samples in the tissue/blood biobank. This study only used this part of BC samples. This study was approved by the Peking University IRB (reference no. IRB00001052-11029).

## Results

### Characteristics of the study population

The epidemiological characteristics of the 1,160 infiltrating ductal BC cases and 1,336 cancer-free controls included in this study were summarized in S1 Table. There was no significant difference in the distribution of age (P = 0.397), BMI (P = 0.661), age at menopause (P = 0.845), menopause status (P = 0.629) and number of childbirth (P = 0.362). As expected, cases were more likely to have an early age at menarche (P = 0.0001), late age at first full-term pregnancy (FFTP) (P<0.0001), and a greater likelihood of family history of cancer in first-degree relatives (P = 0.0064). The clinicopathological parameters of cases were also summarized in S1 Table, these including ER status (Positive, 621 cases; Negative, 237 cases), PR status (Positive, 562 cases; Negative, 292 cases), Her2 status (Positive, 234 cases; Negative, 621 cases), tumor size (≤ 2 cm, 382 cases; > 2 cm, 502 cases), lymph node status (Negative, 457 cases; Positive, 325 cases), clinical stage (0-I, 141 cases; II-IV, 654 cases), chemotherapy status (Yes, 822 cases; No, 144 cases) and endocrine therapy status (Yes, 233 cases; No, 850 cases).

### LD degree between SNPs

**Fig 1:**
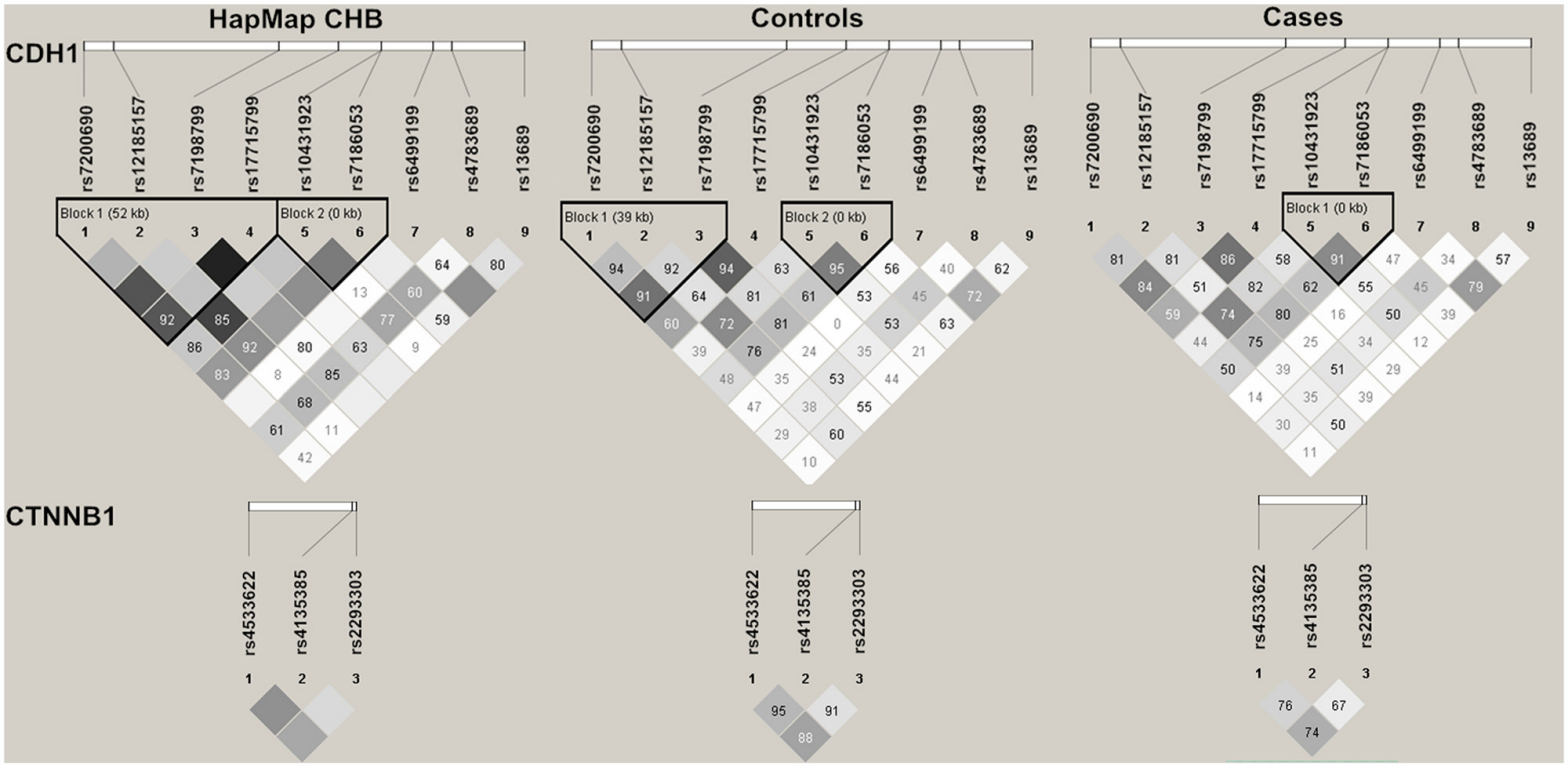
LD maps of htSNPs in HapMap CHB population, controls and breast cancer cases. The values shown in each diamond are the D’×100 (10 means 0.10, 1 means 0.01) and indicate pairwise LD between htSNPs. The dark grey-to-white gradient reflects higher to lower LD values. Dark grey diamonds without a number indicate a complete LD (D’ = 1).

The 12 SNPs were all in agreement with Hardy-Weinberg equilibrium (P>0.05) in the controls (S2 Table). The D’ and r^2^ between 9 SNPs in *CDH1* and between 3 SNPs in *CTNNB1* within our cases, controls and HapMap CHB population were calculated using Haploview 4.2 software. The LD degree of all SNPs in HapMap CHB population, our control and case population were showed in Fig 1. We reconstructed a 3-SNP haplotype block1 [rs7200690 (C>T) + rs12185157 (A>G) + rs7198799 (C>T)] and a 2-SNP haplotype block2 [rs10431923 (T>G) + rs7186053 (G>A)] for *CDH1* according to our genotyping data in controls (Fig 1). While for *CTNNB1*, we didn’t reconstruct any haplotype block due to the weak LD degree of the 3 SNPs in *CTNNB1* in cases, controls as well as in HapMap CHB population (Fig 1).

### Associations of genotypes, haplotypes and diplotypes with BC susceptibility

As shown in Table 1, two-sided x^2^ test only indicated significant differences in allele frequencies between cases and controls for rs2293303 (P = 0.0404) in *CTNNB1* among all the 12 SNPs, but showed significant differences between cases and controls in genotype frequencies of rs7200690, rs7198799, rs17715799 and rs13689 in *CDH1* as well as rs2293303 in *CTNNB1*. Both univariate and multivariate unconditional logistic regression analyses demonstrated that the minor allele homozygotes of these five SNPs could increase BC susceptibility compared with their corresponding heterozygotes and common homozygotes (Table 1). To assess the relative importance of these five at-risk SNPs, multiple logistic regression analysis was performed, which included all the five at-risk SNPs in the full model and used stepwise procedures to select the relatively important SNPs associated with BC risk. After adjusting for the other four SNPs, rs13689 and rs2293303 became much more significant in increasing BC risk (rs13689: aOR = 1.87, 95% CI = 1.25–2.81, P = 0.0024; rs2293303: aOR = 1.88, 95% CI = 1.04–3.40, P = 0.0373), whereas the statistical significance for rs7200690, rs7198799 and rs17715799 disappeared (Table 2).

**Table 1:** Genotype and allele frequencies of the selected SNPs in *CDH1* and *CTNNB1* and their associations with risk of breast cancer.

SNPs	Genotype	Cases (%)	Controls (%)	*P* ^*a*^	*P* ^*b*^	*P*_trend_	OR (95% CI)	*P*	aOR (95% CI) ^c^	*P* ^c^
rs7200690 *CDH1*	CC	744 (64.14)	852 (63.77)	**0.0240**		0.4103	Reference		Reference	
	CT	335 (28.88)	422 (31.59)				0.91 (0.76–1.08)	0.2826	0.91 (0.76–1.08)	0.2736
	TT	81 (6.98)	62 (4.64)				**1.50 (1.06–2.11)**	**0.0221**	**1.50 (1.06–2.12)**	**0.0208**
	T allele frequency	497 (21.42)	546 (20.43)		0.3916					
	CT/TT vs. CC (dominant model)						0.98 (0.84–1.16)	0.8496	0.98 (0.84–1.16)	0.8425
	TT vs. CT/CC (recessive model)						**1.54 (1.10–2.17)**	**0.0126**	**1.55 (1.10–2.18)**	**0.0116**
rs12185157 *CDH1*	AA	337 (29.05)	387 (28.97)	0.6334		0.6277	Reference		Reference	
	AG	540 (46.55)	643 (48.13)				0.91 (0.75–1.11)	0.3395	0.90 (0.74–1.10)	0.3125
	GG	283 (24.40)	306 (22.90)				0.94 (0.76–1.17)	0.5873	0.94 (0.75–1.16)	0.5511
	G allele frequency	1106 (47.67)	1255 (46.97)		0.6193					
	AG/GG vs. AA (dominant model)						0.92 (0.77–1.11)	0.3806	0.92 (0.76–1.10)	0.3489
	GG vs. AG/AA (recessive model)						1.004 (0.84–1.19)	0.9629	0.996 (0.84–1.19)	0.9659
rs7198799 *CDH1*	CC	855 (73.71)	981 (73.43)	**0.0296**		0.4867	Reference		Reference	
	CT	258 (22.24)	423 (24.25)				0.91 (0.76–1.10)	0.3451	0.91 (0.75–1.10)	0.3237
	TT	47 (4.05)	31 (2.32)				**1.74 (1.095–2.76)**	**0.0190**	**1.75 (1.099–2.77)**	**0.0183**
	T allele frequency	352 (15.17)	396 (14.45)		0.4709					
	CT/TT vs. CC (dominant model)						0.99 (0.83–1.18)	0.8749	0.98 (0.82–1.18)	0.8477
	TT vs. CT/CC (recessive model)						**1.78 (1.12–2.82)**	**0.0143**	**1.79 (1.13–2.83)**	**0.0136**
rs17715799 *CDH1*	AA	781 (67.33)	872 (65.27)	**0.0113**		0.9953	Reference		Reference	
	AT	302 (26.03)	403 (30.16)				0.84 (0.70–1.10)	0.0592	0.83 (0.70–1.10)	0.0549
	TT	77 (6.64)	61 (4.57)				**1.41 (0.99–2.00)**	**0.0544**	**1.42 (0.998–2.001)**	**0.0513**
	T allele frequency	456 (19.66)	525 (19.65)		0.9951					
	AT/TT vs. AA (dominant model)						0.91 (0.77–1.08)	0.2789	0.91 (0.77–1.08)	0.2677
	TT vs. AT/AA (recessive model)						**1.49 (1.05–2.10)**	**0.0245**	**1.50 (1.06–2.11)**	**0.0227**
rs10431923 *CDH1*	TT	383 (38.02)	438 (32.78)	0.8221		0.8292	Reference		Reference	
	GT	544 (46.90)	641 (47.98)				0.94 (0.76–1.16)	0.5392	0.93 (0.75–1.15)	0.4851
	GG	233 (20.09)	257 (19.24)				0.96 (0.77–1.21)	0.7516	0.96 (0.77–1.20)	0.7075
	G allele frequency	1010 (43.53)	1155 (43.23)		0.8264					
	GT/GG vs. TT (dominant model)						0.95 (0.78–1.16)	0.5937	0.94 (0.77–1.15)	0.5395
	GG vs. GT/TT (recessive model)						1.01 (0.86–1.20)	0.9017	1.01 (0.85–1.20)	0.9053
rs7186053 *CDH1*	GG	602 (51.90)	667 (49.93)	0.0579		0.9724	Reference		Reference	
	AG	429 (36.98)	548 (41.02)				0.88 (0.73–1.03)	0.0962	0.86 (0.73–1.02)	0.0842
	AA	129 (11.12)	121 (9.06)				1.18 (0.90–1.55)	0.2291	1.19 (0.91–1.56)	0.2082
	A allele frequency	687 (29.61)	790 (29.57)		0.9715					
	AG/AA vs. GG (dominant model)						0.92 (0.79–1.08)	0.3259	0.92 (0.79–1.10)	0.3102
	AA vs. AG/GG (recessive model)						1.26 (0.97–1.63)	0.0872	1.27 (0.98–1.65)	0.0742
rs6499199 *CDH1*	CC	888 (76.55)	1028 (76.95)	0.2594		0.4872	Reference		Reference	
	CT	241 (20.78)	285 (21.33)				0.98 (0.81–1.19)	0.8295	0.99 (0.82–1.20)	0.9230
	TT	31 (2.67)	23 (1.72)				1.56 (0.90–2.70)	0.1110	1.58 (0.91–2.73)	0.1030
	T allele frequency	303 (13.06)	331 (12.39)		0.4766					
	CT/TT vs. CC (dominant model)						1.02 (0.85–1.23)	0.8195	1.03 (0.86–1.25)	0.7230
	TT vs. CT/CC (recessive model)						1.57 (0.91–2.70)	0.1063	1.58 (1.001–1.49)	0.1004
rs4783689 *CDH1*	CC	576 (49.66)	646 (48.35)	0.8095		0.5694	Reference		Reference	
	CT	465 (40.09)	550 (41.17)				0.95 (0.80–1.12)	0.5323	0.95 (0.80–1.12)	0.5069
	TT	119 (10.26)	140 (10.48)				0.95 (0.73–1.25)	0.7274	0.95 (0.72–1.24)	0.6913
	T allele frequency	703 (30.30)	830 (31.06)		0.5609					
	CT/TT vs. CC (dominant model)						0.95 (0.81–1.11)	0.5164	0.95 (0.81–1.11)	0.4846
	TT vs. CT/CC (recessive model)						0.98 (0.75–1.26)	0.8572	0.97 (0.75–1.26)	0.8263
rs13689 *CDH1*	TT	766 (66.03)	875 (65.49)	**0.0047**		0.4013	Reference		Reference	
	CT	330 (28.45)	420 (31.44)				0.90 (0.75–1.07)	0.2226	0.90 (0.76–1.08)	0.2586
	CC	64 (5.52)	41 (3.07)				**1.78 (1.19–2.67)**	**0.0050**	**1.83 (1.22–2.75)**	**0.0033**
	C allele frequency	458 (19.74)	502 (18.79)		0.3937					
	CT/CC vs. TT (dominant model)						0.98 (0.83–1.15)	0.7766	0.98 (0.83–1.16)	0.8013
	CC vs. CT/TT (recessive model)						**1.84 (1.24–2.75)**	**0.0027**	**1.89 (1.27–2.83)**	**0.0018**
rs4533622 *CTNNB1*	CC	725 (62.50)	817 (61.15)	0.6557		0.6748	Reference		Reference	
	AC	366 (31.55)	444 (33.23)				0.93 (0.78–1.10)	0.3979	0.93 (0.78–1.10)	0.3890
	AA	69 (5.95)	75 (5.61)				1.04 (0.74–1.46)	0.8357	1.05 (0.74–1.47)	0.7940
	A allele frequency	504 (21.72)	594 (22.23)		0.6666					
	AC/AA vs. CC (dominant model)						0.95 (0.80–1.11)	0.4899	0.95 (0.80–1.11)	0.4922
	AA vs. AC/CC (recessive model)						1.60 (0.76–1.49)	0.7203	1.07 (0.79–1.51)	0.6781
rs4135385 *CTNNB1*	GG	295 (25.43)	356 (26.65)	0.7748		0.6438	Reference		Reference	
	AG	601 (51.81)	677 (50.67)				1.07 (0.89–1.30)	0.4765	1.08 (0.89–1.31)	0.4318
	AA	264 (22.76)	303 (22.68)				1.05 (0.84–1.32)	0.6639	1.06 (0.85–1.33)	0.5947
	A allele frequency	1129 (48.66)	1283 (48.02)		0.6480					
	AG/AA vs. GG (dominant model)						1.07 (0.89–1.27)	0.4908	1.07 (0.90–1.29)	0.4350
	AA vs. AG/GG (recessive model)						1.01 (0.83–1.21)	0.9625	1.01 (0.84–1.22)	0.9098
rs2293303 *CTNNB1*	CC	879 (75.78)	1048 (78.44)	0.0629		**0.0444**	Reference		Reference	
	TC	251 (21.64)	269 (20.13)				1.11 (0.92–1.35)	0.2814	1.11 (0.91–1.35)	0.2961
	TT	30 (2.59)	19 (1.42)				**1.88 (1.05–3.37)**	**0.0330**	**1.92 (1.07–3.43)**	**0.0288**
	T allele frequency	311 (13.41)	307 (11.49)		**0.0404**					
	TC/TT vs. CC (dominant model)						1.16 (0.97–1.40)	0.1133	1.16 (0.97–1.40)	0.1113
	TT vs. TC/CC (recessive model)						**1.94 (1.08–3.51)**	**0.0272**	**1.97 (1.09–3.55)**	**0.0246**

^a^ Two-sided χ2 test for difference in frequency distribution of genotypes between cases and controls.

^b^ Two-sided χ2 test for difference in frequency distribution of alleles between cases and controls.

^c^ Adjusted for age at menarche, age of first birth and family history of cancer in first-degree relatives.

Bold numbers indicate a statistical significance at 0.05 level.

**Table 2:** Multiple logistic regression analyses including all the 5 at-risk SNPs in full model.

Gene	SNP	OR (95% CI)	*P*	aOR (95% CI) ^a^	*P* ^a^
*CDH1*	rs7200690 (recessive)	1.17 (0.75–1.81)	0.4961	1.16 (0.74–1.79)	0.5221
	rs7198799 (recessive)	1.31 (0.70–2.45)	0.3976	1.32 (0.70–2.47)	0.3881
	rs17715799 (recessive)	1.26 (0.85–1.89)	0.2496	1.27 (0.85–1.90)	0.2353
	rs13689 (recessive)	1.82 (1.22–2.73)	**0.0036**	1.87 (1.25–2.81)	**0.0024**
*CTNNB1*	rs2293303 (recessive)	1.86 (1.03–3.36)	**0.0410**	1.88 (1.04–3.40)	**0.0373**

^a^ Adjusted for age at menarche, age of first birth and family history of cancer in first-degree relatives.

Bold numbers indicate a statistical significance at 0.05 level.

As haplotype analysis can capture more polymorphisms than genotype analysis, we examined the associations between haplotypes in *CDH1* and BC risk. Neither the 3-SNP haplotypes in block1 nor the 2-SNP haplotypes in block2 were associated with BC risk based on x^2^ test and logistic regression analysis. However, the 3-SNP haplotype pairs (diplotype) CGC/TGC [rs7200690 (C>T) + rs12185157 (A>G) + rs7198799 (C>T)], could increase BC risk compared with common diplotype CAC/CAC (aOR = 1.59, 95% CI = 1.01–2.52, P = 0.0472) (S3 Table).

For gene-gene interaction, the GMDR analysis indicated that, with adjustment for covariates (age, BMI, age at menarche, age of first birth and family history of cancer in first-degree relatives), the 2-order model including rs17715799 and rs13689 would be the best model for predicting BC risk, which had high Cross Validation Consistency (8/10) and Testing Balanced Accuracy (0.5165) with the Sign Test P = 0.0107 (Table 3). The multiple logistic regression analysis demonstrated that women harboring rs17715799 TT and rs13689 CC had a significantly higher risk of BC after adjustment for age, BMI, age at menarche, age of first birth and family history of cancer in first-degree relatives (OR = 1.68, 95% CI = 1.28–2.20, P = 0.0002) (Table 4). For gene-environment interaction, after adjustment for age, BMI, age at menarche, age of first birth and family history of cancer in first-degree relatives, the best model included rs13689 and menarche-FFTP interval with the greatest Cross Validation Consistency (10/10) and Testing Balanced Accuracy (0.5955) (Sign Test P = 0.0010), indicating that the interaction of rs13689 and menarche-FFTP interval play an important role on BC risk (Table 3). Notably, menarche-FFTP interval was the best one-order model and emerged in all five models, suggesting that menarche-FFTP interval was an extremely important factor affecting BC susceptibility. Therefore, we further performed logistic regression analysis to assess the joint effect of these at-risk SNPs and menarche-FFTP interval on BC risk. The results revealed that women harboring the minor allele homozygotes of one of the five at-risk SNPs and longer menarche-FFTP interval (>11 years) had a remarkable increase in BC risk (all P<0.05), indicating a synergistic effect of these at-risk SNPs and menarche-FFTP interval on BC susceptibility (Table 5).

**Table 3:** Comparison of the best models identified by GMDR for gene-gene and gene-environment interactions.

Best model ^a^	Training balanced accuracy ^b^	Testing balanced accuracy ^b^	Cross-validation consistency ^b^	Sign test (P) ^b^
Gene-gene				
x9	0.5132	0.5067	8/10	8 (0.0547)
x4 x9	0.5236	0.5165	8/10	9 (0.0107)
x4 x6 x9	0.5293	0.5194	5/10	9 (0.0107)
x4 x9 x10 x11	0.5341	0.5133	6/10	7 (0.1719)
x3 x4 x9 x10 x11	0.5384	0.5082	3/10	6 (0.3770)
Gene-environment				
y1*	0.5909	0.5909	10/10	10 (0.0010)
x9 y1*	0.5955	0.5955	10/10	10 (0.0010)
x1 x3 y1*	0.5986	0.5782	3/10	10 (0.0010)
x2 x3 x6 y1*	0.6085	0.5808	3/10	10 (0.0010)
x2 x4 x6 x7 y1*	0.6253	0.5737	4/10	10 (0.0010)

^a^ The x1 x2 x3 x4 x6 x7 x9 x10 x11 y1 represent rs7200690, rs12185157, rs7198799, rs17715799, rs7186053, rs6499199, rs13689, rs4533622, rs4135385 and Menarche-FFTP interval (> 11 years vs ≤11 years) respectively.

* Both parous and nulliparous women were included. This parameter was calculated by age at FFTP minus age at menarche in all parous women, age at menopause minus age at menarche in postmenopausal nulliparous women, and age at breast tumor onset (case) or age at interview (control) minus age at menarche in premenopausal nulliparous women.

^b^ Adjusted for age, BMI, age at menarche, age of first birth and family history of cancer in first-degree relatives.

**Table 4:** Multiple logistic regression analysis of the major risk factors for BC.

Risk factors	OR (95% CI)	*P*
Age	1.03 (0.88–1.21)	0.7137
BMI	1.19 (1.00–1.42)	0.0478
Age at menarche	1.80 (0.93–3.50)	0.0815
Age of first birth	2.13 (0.98–4.63)	0.0562
Family history of cancer	1.23 (1.01–1.50)	0.0396
rs17715799 TT and rs13689 CC	1.72 (1.31–2.26)	< .0001

**Table 5:** Risk of breast cancer associated with the combination of susceptible SNPs and menarche-FFTP interval.

			Menarche-FFTP interval ≤11 years			Menarche-FFTP interval > 11 years		
Gene	SNP/Genotype		Case /Control	aOR (95% CI) ^a^	*P* ^a^	Case/Control	aOR (95% CI) ^a^	*P* ^a^
*CDH1*	rs7200690	CC+CT	479/808	Reference		600/466	2.18 (1.85–2.57)	**<.0001**
		TT	32/35	1.55 (0.95–2.53)	0.0818	49/27	3.07 (1.90–4.98)	**<.0001**
	rs7198799	CC+CT	495/824	Reference		618/481	2.15 (1.82–2.53)	**<.0001**
		TT	16/19	1.41 (0.72–2.76)	0.3205	31/12	4.31 (2.20–8.48)	**<.0001**
	rs17715799	AA+AT	483/803	Reference		600/472	2.12 (1.80–2.50)	**<.0001**
		TT	28/40	1.17 (0.71–1.92)	0.5381	49/21	3.89 (2.31–6.57)	**<.0001**
	rs13689	TT+CT	478/816	Reference		618/479	2.21 (1.88–2.61)	**<.0001**
		CC	33/27	2.10 (1.24–3.53)	**0.0054**	31/14	3.80 (2.00–7.21)	**<.0001**
*CTNNB1*	rs2293303	CC+TC	498/832	Reference		632/486	2.18 (1.86–2.57)	**<.0001**
		TT	13/11	1.98 (0.88–4.46)	0.0980	17/7	4.07 (1.68–9.87)	**0.0019**

^a^ Adjusted for family history of cancer in first-degree relatives. Bold numbers indicate a statistical significance at 0.05 level.

### Associations of genotypes and haplotypes with event-free survival

**Fig 2:**
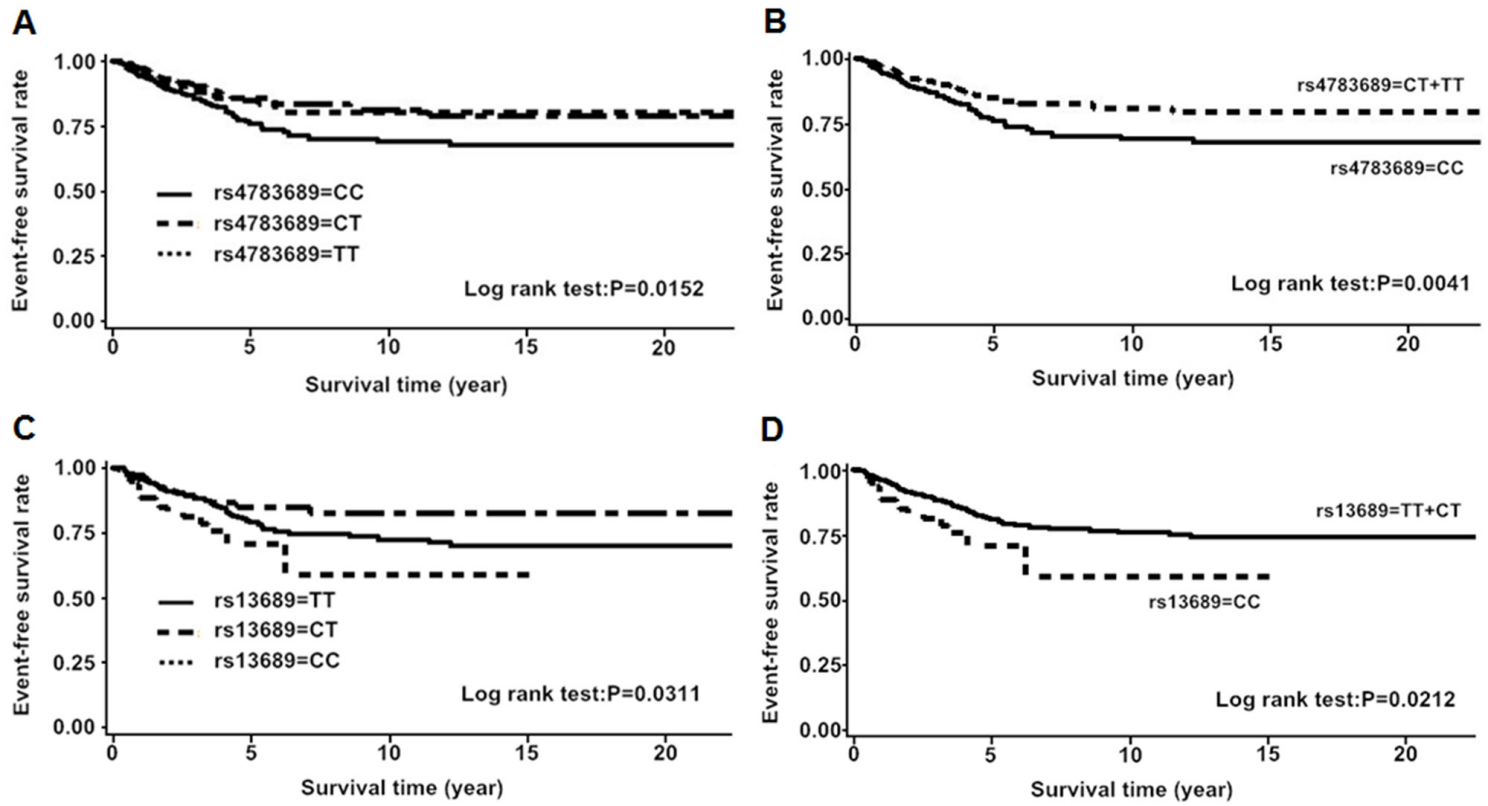
Kaplan-Meier estimates of event-free survival according to SNP rs4783689 and rs13689. A and B for rs4783689; C and D for rs13689.

First, we analyzed the associations of genotype, haplotype and diplotype with BC event-free survival. In *CDH1*, the patients with CT or TT genotype of rs4783689 (C>T) were more likely to have favorable event-free survival compared to the CC genotype carriers (HR = 0.63, 95% CI = 0.46–0.87, P = 0.0045). The minor allele homozygotes of rs13689 (T>C) were correlated with worse event-free survival (HR = 1.85, 95% CI = 1.09–3.15, P = 0.0233). Furthermore, the haplotype TGC [rs7200690 (C>T) + rs12185157 (A>G) + rs7198799 (C>T)], was correlated with an unfavorable event-free survival when compared to the most common haplotype CAC (HR = 1.49, 95% CI = 1.02–2.18, P = 0.0380). However, the statistical significance for rs4783689, rs13689 and haplotype TGC disappeared after adjusting for clinicopathological parameters (rs4783689: aHR = 0.75, 95% CI = 0.44–1.27, P = 0.2863; rs13689: aHR = 0.62, 95% CI = 0.15–2.54, P = 0.5043; haplotype TGC: aHR = 1.36, 95% CI = 0.71–2.59,P = 0.3558) (Fig 2, S4 Table). In *CTNNB1*, no genotypes, haplotypes and diplotypes were associated with event-free survival (data not shown).

**Fig 3:**
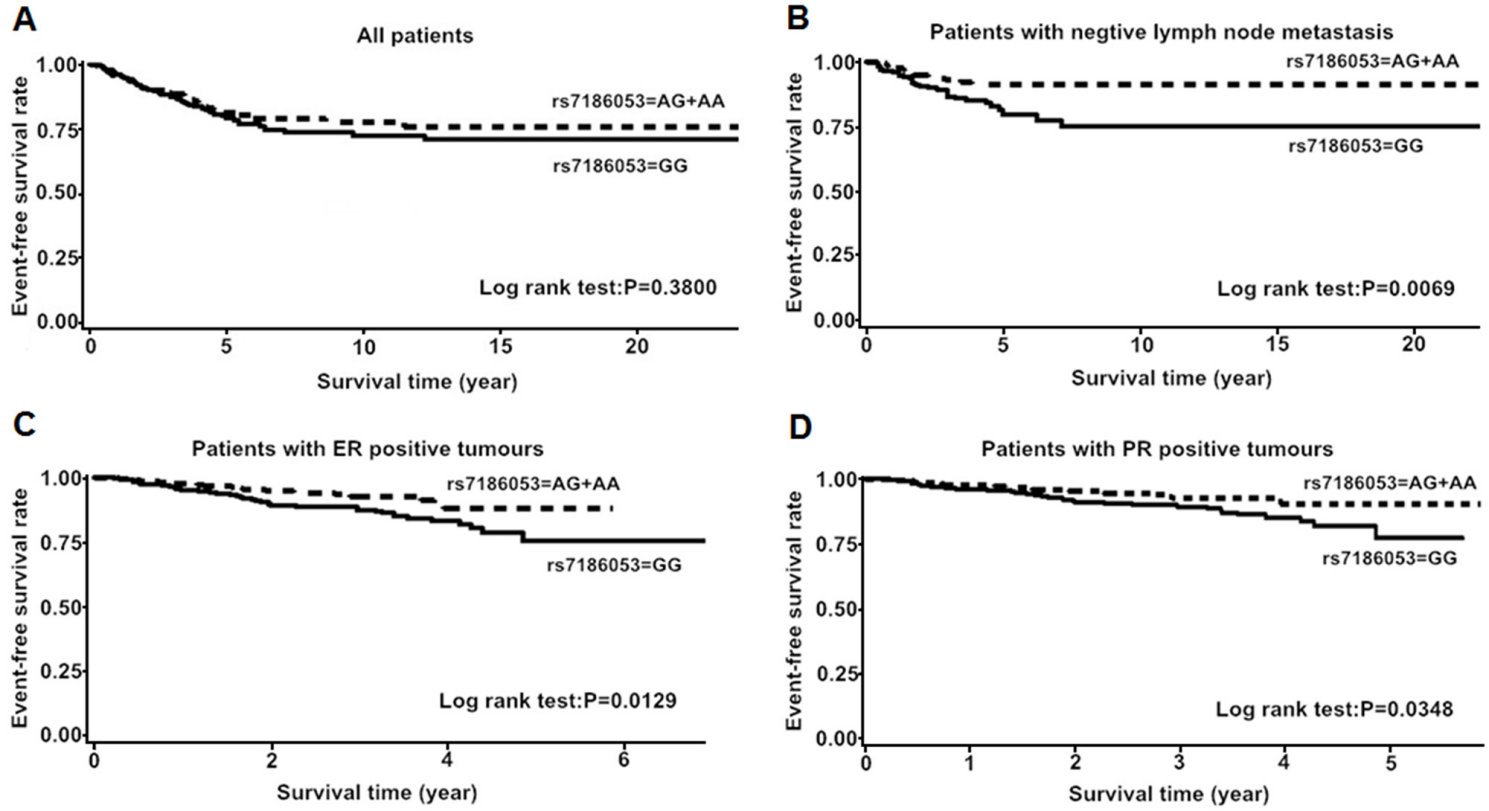
Kaplan-Meier estimates of event-free survival according to rs7186053 genotypes. A: among all breast cancer patients; B: among patients without lymph node metastasis; C: among patients with ER-positive tumors; D: among patients with PR-positive tumors. P values were calculated by log rank test.

**Fig 4:**
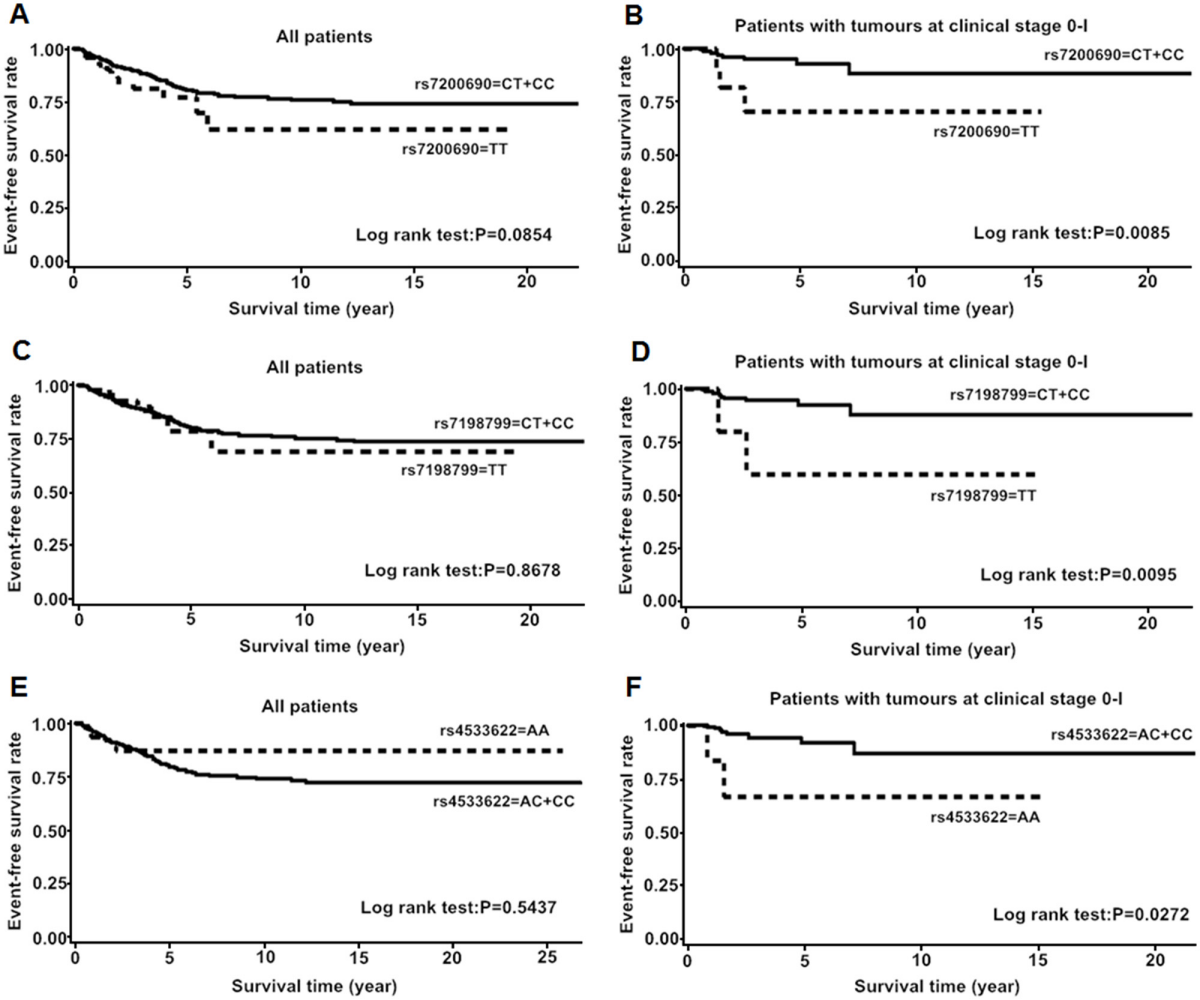
Kaplan-Meier estimates of event-free survival among all patients and patients with stage 0-I tumors respectively. A and B for rs7200690; C and D for rs7198799; E and F for rs4533622. P values were calculated by log rank test.

Then, we performed stratified analysis. In *CDH1*, the patients harboring AA or GA genotype of rs7186053 (G>A) had favorable event-free survival in less aggressive tumor subgroups, such as in ER-positive group, PR-positive group and negative lymph node metastasis group (Fig 3, Table 6). The minor allele homozygote carriers of rs7200690 (C>T) or rs7198799 (C>T) had unfavorable event-free survival among patients with clinical stage 0-I tumors (Fig 4, Table 7). In *CTNNB1*, AA genotype of rs4533622 (C>A) was associated with worse BC event-free survival among patients with clinical stage 0-I tumors (aHR = 9.04, 95% CI = 0.93–87.96, P = 0.0580) (Fig 4, Table 7).

**Table 6:** Stratified event-free survival analysis of rs7186053 by ER, PR and lymph node status.

Variables	No_event_ / No (%)		HR (95%CI)	*P*	aHR (95%CI) *	*P* *
	GG	AG/AA				
ER						
Positive	47/315 (15)	20/269 (7)	0.52 (0.30–0.87)	**0.0146**	0.29 (0.12–0.67)	**0.0039**
Negative	13/112 (12)	20/108 (19)	1.75 (0.86–3.54)	0.1173	2.60 (1.05–6.38)	0.0372
PR						
Positive	37/284 (13)	16/242 (7)	0.53 (0.29–0.96)	**0.0379**	0.42 (0.18–1.00)	**0.0510**
Negative	23/141 (16)	24/133 (18)	1.16 (0.65–2.07)	0.5930	1.28 (0.59–2.74)	0.5265
Lymph node metastasis						
Negative	37/242 (15)	15/206 (7)	0.44 (0.24–0.81)	**0.0086**	0.35 (0.13–0.95)	**0.0397**
Positive	45/162 (28)	43/157 (27)	1.17 (0.76–1.78)	0.4650	1.08 (0.53–2.18)	0.8203

*Adjusted for ER status, PR status, Her2 status, tumor size, clinical stage, lymphnode metastasis, chemotherapy and endocrine therapy except for stratified factors. Bold numbers indicate a statistical significance at 0.05 level.

**Table 7:** Stratified event-free survival analysis of rs rs7200690, rs7198799 in *CDH1* and rs4533622 in *CTNNB1* by clinical stage.

	No_event_ /No (%)					
Clinical stage	rs7200690 CC/CT	rs7200690 TT	HR (95%CI)	*P*	aHR (95%CI) *	*P* *
0-I	8/126 (6)	3/11 (27)	5.05 (1.32–19.27)	**0.0179**	10.30 (1.42–74.73)	**0.0211**
II-IV	109/578 (19)	9/40 (23)	1.33 (0.67–2.63)	0.4112	1.37 (1.68–2.70)	0.2111
Clinical stage	rs7198799 CC/CT	rs7198799 TT				
0-I	9/132 (7)	2/5 (40)	5.97 (1.29–27.70)	**0.0226**	10.91 (1.13–105.34)	**0.0389**
II-IV	115/595 (19)	3/23 (13)	0.71 (0.23–2.24)	0.5622	0.80 (0.26–2.29)	0.4322
Clinical stage	rs4533622 CC/CA	rs4533622 AA				
0-I	9/131 (7)	2/6 (33)	4.84 (1.03–22.70)	**0.0456**	9.04 (0.93–87.96)	**0.0580**
II-IV	113/574 (20)	5/44 (11)	0.59 (0.24–1.45)	0.2530	1.02 (0.37–2.82)	0.9721

* Adjusted for ER status, PR status, Her2 status, tumor size, clinical stage, lymphnode metastasis, chemotherapy and endocrine therapy.

Bold numbers indicate a statistical significance at 0.05 level.

## Discussion

To our knowledge, this is the first haplotype-based association study of *CDH1* and *CTNNB1* with breast cancer susceptibility and patients’ survival in Chinese Han population.

As *CDH1* mutations were frequent in gastric cancer (GC) [32, 33], several studies were performed to examine the association of *CDH1* genetic polymorphisms with GC risk [34–36]. Zhan Z et al. genotyped four potentially functional polymorphisms (rs13689, rs1801552, rs16260 and rs17690554) of the *CDH1* gene in a case-control study of 387 gastric cancer cases and 392 controls, and they found no association of these four SNPs with overall gastric cancer risk, however, they revealed that rs16260 and rs17690554 were associated with the risk of diffuse gastric cancer in subgroup analysis [36]. Another two studies performed by Cui Y et al. and Zhang XF respectively did not find any association between rs16260 and gastric cancer risk [34, 35]. In this study, we found that rs7200690 (C>T), rs7198799 (C>T), rs17715799 (A>T) and rs13689 (T>C) conferred around 50%, 80%, 50% and 90% increased risk for BC respectively in recessive model. Alicia Beeghly-Fadiel and colleagues genotyped 40 SNPs of *CDH1* in 2,083 BC cases and 2,152 controls from urban Shanghai. They demonstrated that BC risk was not only associated with rs13689, but also associated with rs2059254 and rs12919719, which were in high LD with rs7198799 and rs17715799 respectively [14]. Therefore, their results are consistent with ours. A functional polymorphism in *CDH1* promoter (rs16260, −160 C/A), which was reported to reduce E-cadherin expression by the minor allele A [12], was found to be linked with 30% increased risk of BC [13]. However, no effect of rs16260 was seen on BC risk in a European population-based study as well as on BC survival in a British population-based study [37, 38]. Although rs16260 was not directly genotyped in our study, the genetic variation of this polymorphism was captured. The SNP rs7200690, genotyped in our study, is reported to be in perfect LD (D’ = 1.0, r^2^ = 1.0) with rs16260 [39]. In the present study, rs7200690 was demonstrated to be associated with BC risk. Notably, the minor allele homozygotes of rs7200690 (C>T) was shown to be associated with worse BC event-free survival among patients with clinical stage 0-I tumors. Statistical differences remained significant after adjustment for other survival affecting factors. The htSNP rs7200690 tagged 19 SNPs (r^2^ = 1.0), these including a functional SNP rs16260 in *CDH1* promoter and 18 intron SNPs. Therefore, we speculated that the association of rs7200690 with BC was most likely driven by its tagged SNPs rs16260. So, an additional fine-mapping study and the corresponding functional study will be helpful in identifying the causal variants. In addition, we found that rs13689, located in 3’UTR of *CDH1*, was associated with increased risk and unfavorable survival of BC. We also found that rs13689 interacted with rs17715799 and menarche-FFTP interval, and these factors jointly affected BC risk. 3’UTR is essential in mRNA stability and localization [28], and it may also be the binding site of miRNA. Polymorphisms in the 3’UTRs of several genes have been reported to be associated with diseases by affecting miRNA-regulated gene/protein expression [40]. Therefore, functional studies are needed to determine how this SNP rs13689 influence the BC susceptibility and event-free survival.

Given the combined effect of multiple alleles may provide a stronger predictor than individual SNPs, we therefore investigated disease associations with haplotypes and diplotypes. The 3-SNPs diplotype CGC/TGC [rs7200690 (C>T) + rs12185157 (A>G) + rs7198799 (C>T)], could increase about 60% of BC risk. Moreover, haplotype TGC was correlated with an unfavorable event-free survival.

For *CTNNB1*, we couldn’t reconstruct haplotype, so, we just conducted genotype analysis. We found that rs2293303 conferred a 1.0-fold increased risk of BC in recessive model. Wang et al. analyzed five tagSNPs of *CTNNB1* in 944 gastric cancer cases and 848 controls in Chinese population, and found that rs2293303 was correlated with increased risk of gastric cancer [41]. It indicates that rs2293303 may play a similar role on breast and gastric cancer susceptibility in Chinese population. Importantly, rs2293303, located in gene-coding regions of *CTNNB1*, is a synonymous SNP (sSNPs). Although sSNPs do not change the amino acid composition of the encoded proteins owing to the degeneracy of the genetic code, but considerable evidence has accumulated to show that synonymous substitutions could affect mRNA splicing, mRNA stability, splicing accuracy, mRNA structure, translation fidelity and thus protein expression and enzymatic activity [42]. In addition, sSNPs can also affect protein folding and conformation because codon bias could affect tertiary protein structure [42], therefore, they have functional and clinical consequences. Additional mechanism studies are needed to determine how the SNP rs2293303 influence the BC susceptibility. Alanazi and colleagues genotyped rs13072632 and rs4135385 in *CTNNB1* in 99 cases and 93 controls in Suadi population, and found that rs4135385 was linked with increased BC risk [24]. Lee et al. analyzed 1,536 SNPs in 203 genes among 209 cases and 209 controls in Korean women, and indicated that rs4135385 was associated with decreased BC risk [25]. In our study, no association was found between rs4135385 and BC risk. The discrepancies among these results could be due to the limited sample size of each study, ethnic diversity of populations and complicated environmental factors. Mostowska el at. genotyped SNP rs4533622 and rs2953 of *CTNNB1* in 228 ovarian cancer women and 282 controls, and found no association between the two SNPs and ovarian cancer risk [43]. Similarly, no association between rs4533622 and BC risk was found in the present study. Ting and colleagues genotyped 10 tagSNPs of *CTNNB1* and *APC* in 282 Chinese colorectal cancer patients, and found no associations between the analyzed SNPs and colorectal cancer survival [44]. Wang el at. demonstrated that rs4135385 AG/AA genotypes were significantly associated with a favorable gastric cancer survival [41]. In our event-free survival analysis, we identified no association between the three SNPs of *CTNNB1* and BC event-free survival. However, the stratified analysis demonstrated that rs4533622 was associated with worse BC event-free survival among patients with clinical stage 0-I tumors.

Breast cancer is a complex disease, resulting from the interaction of multiple environmental, hormonal, and lifestyle risk factors with the individual’s genetic factors [45]. We, therefore, analyzed the gene-gene and gene-environment interactions on BC susceptibility using GMDR method and logistic regression analysis. We observed interactions on BC risk between rs17715799 and rs13689 as well as rs13689 and menarche-FFTP interval. Furthermore, the five at-risk SNPs and longer menarche-FFTP interval (>11 years) would jointly affect BC susceptibility. All these results suggested some putative interactions between gene-gene and gene-environment on BC susceptibility. In summary, this study indicated that the genetic polymorphisms of *CDH1* and *CTNNB1* were associated with breast cancer susceptibility and prognosis. Due to the fact that these SNPs examined in this study were htSNPs of the two genes, additional studies are warranted to verify these results and identify the causal variants.

## Supporting Information

S1 Table Characteristics of breast cancer cases and cancer-free controls. (DOC)

S2 Table Hardy-Weinberg equilibrium analysis of the SNPs in *CDH1* and *CTNNB1*. (DOC)

S3 Table Frequencies of diplotype of block1 in *CDH1* and its association with risk of breast cancer. (DOC)

S4 Table Univariate and Multivariate Cox proportional hazard analysis of the SNPs and haplotypes in *CDH1* and *CTNNB1* in relation to event-free survival of breast cancer patients. (DOC)

## References

[1] Ferlay J, Soerjomataram I, Dikshit R, Eser S, Mathers C, Rebelo M, et al Cancer incidence and mortality worldwide: Sources, methods and major patterns in GLOBOCAN 2012. International journal of cancer Journal international du cancer. 2014 9 13 . 25220842 10.1002/ijc.29210

[2] Christiansen JJ, Rajasekaran AK. Reassessing epithelial to mesenchymal transition as a prerequisite for carcinoma invasion and metastasis. Cancer research. 2006 9 1;66(17):8319–26. . 16951136 10.1158/0008-5472.CAN-06-0410

[3] Micalizzi DS, Farabaugh SM, Ford HL. Epithelial-mesenchymal transition in cancer: parallels between normal development and tumor progression. Journal of mammary gland biology and neoplasia. 2010 6;15(2):117–34. Pubmed Central PMCID: 2886089. 10.1007/s10911-010-9178-9 20490631 PMC2886089

[4] Iwatsuki M, Mimori K, Yokobori T, Ishi H, Beppu T, Nakamori S, et al Epithelial-mesenchymal transition in cancer development and its clinical significance. Cancer science. 2010 2;101(2):293–9. 10.1111/j.1349-7006.2009.01419.x 19961486 PMC11159985

[5] De Wever O, Pauwels P, De Craene B, Sabbah M, Emami S, Redeuilh G, et al Molecular and pathological signatures of epithelial-mesenchymal transitions at the cancer invasion front. Histochemistry and cell biology. 2008 9;130(3):481–94. Pubmed Central PMCID: 2522326. 10.1007/s00418-008-0464-1 18648847 PMC2522326

[6] Cowin P, Rowlands TM, Hatsell SJ. Cadherins and catenins in breast cancer. Current opinion in cell biology. 2005 10;17(5):499–508. . 16107313 10.1016/j.ceb.2005.08.014

[7] van Roy F, Berx G. The cell-cell adhesion molecule E-cadherin. Cellular and molecular life sciences: CMLS. 2008 11;65(23):3756–88. 10.1007/s00018-008-8281-1 18726070 PMC11131785

[8] Berx G, Van Roy F. The E-cadherin/catenin complex: an important gatekeeper in breast cancer tumorigenesis and malignant progression. Breast cancer research: BCR. 2001;3(5):289–93. . Pubmed Central PMCID: 138690. 11597316 10.1186/bcr309 PMC138690

[9] Berx G, Cleton-Jansen AM, Nollet F, de Leeuw WJ, van de Vijver M, Cornelisse C, et al E-cadherin is a tumour/invasion suppressor gene mutated in human lobular breast cancers. The EMBO journal. 1995 12 15;14(24):6107–15. . Pubmed Central PMCID: 394735. 8557030 10.1002/j.1460-2075.1995.tb00301.x PMC394735

[10] Masciari S, Larsson N, Senz J, Boyd N, Kaurah P, Kandel MJ, et al Germline E-cadherin mutations in familial lobular breast cancer. Journal of medical genetics. 2007 11;44(11):726–31. . Pubmed Central PMCID: 2752184. 17660459 10.1136/jmg.2007.051268 PMC2752184

[11] Xie ZM, Li LS, Laquet C, Penault-Llorca F, Uhrhammer N, Xie XM, et al Germline mutations of the E-cadherin gene in families with inherited invasive lobular breast carcinoma but no diffuse gastric cancer. Cancer. 2011 7 15;117(14):3112–7. 10.1002/cncr.25876 21271559

[12] Li LC, Chui RM, Sasaki M, Nakajima K, Perinchery G, Au HC, et al A single nucleotide polymorphism in the E-cadherin gene promoter alters transcriptional activities. Cancer research. 2000 2 15;60(4):873–6. . 10706097

[13] Yu JC, Hsu HM, Chen ST, Hsu GC, Huang CS, Hou MF, et al Breast cancer risk associated with genotypic polymorphism of the genes involved in the estrogen-receptor-signaling pathway: a multigenic study on cancer susceptibility. Journal of biomedical science. 2006 5;13(3):419–32. . 16502042 10.1007/s11373-006-9069-7

[14] Beeghly-Fadiel A, Lu W, Gao YT, Long J, Deming SL, Cai Q, et al E-cadherin polymorphisms and breast cancer susceptibility: a report from the Shanghai Breast Cancer Study. Breast cancer research and treatment. 2010 6;121(2):445–52. Pubmed Central PMCID: 2874636. 10.1007/s10549-009-0579-7 19834798 PMC2874636

[15] Logan CY, Nusse R. The Wnt signaling pathway in development and disease. Annual review of cell and developmental biology. 2004;20:781–810. . 15473860 10.1146/annurev.cellbio.20.010403.113126

[16] Karabacak T, Egilmez R, Arpaci RB, Pfeiffer ES. Beta-catenin expression in in situ and infiltrative ductal carcinomas of the breast. Turk patoloji dergisi. 2011;27(3):185–8. 10.5146/tjpath.2011.01073 21935866

[17] Abd El-Rehim D, Ali MM. Aberrant expression of beta-catenin in invasive ductal breast carcinomas. Journal of the Egyptian National Cancer Institute. 2009 6;21(2):185–95. . 21057570

[18] Fanelli MA, Montt-Guevara M, Diblasi AM, Gago FE, Tello O, Cuello-Carrion FD, et al P-cadherin and beta-catenin are useful prognostic markers in breast cancer patients; beta-catenin interacts with heat shock protein Hsp27. Cell stress & chaperones. 2008 Summer;13(2):207–20. . Pubmed Central PMCID: 2673888. 18320359 10.1007/s12192-007-0007-z PMC2673888

[19] Kizildag S, Zengel B, Vardar E, Sakizli M. beta-catenin gene mutation in invasive ductal breast cancer. Journal of BUON: official journal of the Balkan Union of Oncology. 2008 Oct-Dec;13(4):533–6. . 19145675

[20] Ueda M, Gemmill RM, West J, Winn R, Sugita M, Tanaka N, et al Mutations of the beta- and gamma-catenin genes are uncommon in human lung, breast, kidney, cervical and ovarian carcinomas. British journal of cancer. 2001 7 6;85(1):64–8. . Pubmed Central PMCID: 2363927. 11437403 10.1054/bjoc.2001.1863 PMC2363927

[21] Moreno-Bueno G, Hardisson D, Sanchez C, Sarrio D, Cassia R, Garcia-Rostan G, et al Abnormalities of the APC/beta-catenin pathway in endometrial cancer. Oncogene. 2002 11 14;21(52):7981–90. . 12439748 10.1038/sj.onc.1205924

[22] Saegusa M, Okayasu I. Frequent nuclear beta-catenin accumulation and associated mutations in endometrioid-type endometrial and ovarian carcinomas with squamous differentiation. The Journal of pathology. 2001 5;194(1):59–67. . 11329142 10.1002/path.856

[23] Grigoryan T, Wend P, Klaus A, Birchmeier W. Deciphering the function of canonical Wnt signals in development and disease: conditional loss- and gain-of-function mutations of beta-catenin in mice. Genes & development. 2008 9 1;22(17):2308–41. . Pubmed Central PMCID: 2749675. 18765787 10.1101/gad.1686208 PMC2749675

[24] Alanazi MS, Parine NR, Shaik JP, Alabdulkarim HA, Ajaj SA, Khan Z. Association of single nucleotide polymorphisms in Wnt signaling pathway genes with breast cancer in Saudi patients. PloS one. 2013;8(3):e59555 Pubmed Central PMCID: 3597615. 10.1371/journal.pone.0059555 23516639 PMC3597615

[25] Lee JY, Park AK, Lee KM, Park SK, Han S, Han W, et al Candidate gene approach evaluates association between innate immunity genes and breast cancer risk in Korean women. Carcinogenesis. 2009 9;30(9):1528–31. 10.1093/carcin/bgp084 19372141

[26] Gabriel SB, Schaffner SF, Nguyen H, Moore JM, Roy J, Blumenstiel B, et al The structure of haplotype blocks in the human genome. Science. 2002 6 21;296(5576):2225–9. . 12029063 10.1126/science.1069424

[27] Wang H, Xie YT, Han JY, Ruan Y, Song AP, Zheng LY, et al Genetic polymorphisms in centrobin and Nek2 are associated with breast cancer susceptibility in a Chinese Han population. Breast cancer research and treatment. 2012 11;136(1):241–51. 10.1007/s10549-012-2244-9 23001753

[28] Li Y, Chen YL, Xie YT, Zheng LY, Han JY, Wang H, et al Association study of germline variants in CCNB1 and CDK1 with breast cancer susceptibility, progression, and survival among Chinese Han women. PloS one. 2013;8(12):e84489 Pubmed Central PMCID: 3873991. 10.1371/journal.pone.0084489 24386390 PMC3873991

[29] Han JY, Wang H, Xie YT, Li Y, Zheng LY, Ruan Y, et al Association of germline variation in CCNE1 and CDK2 with breast cancer risk, progression and survival among Chinese Han women. PloS one. 2012;7(11):e49296 Pubmed Central PMCID: 3504019. 10.1371/journal.pone.0049296 23185313 PMC3504019

[30] Ruan Y, Song AP, Wang H, Xie YT, Han JY, Sajdik C, et al Genetic polymorphisms in AURKA and BRCA1 are associated with breast cancer susceptibility in a Chinese Han population. The Journal of pathology. 2011 12;225(4):535–43. 10.1002/path.2902 21598251

[31] Barrett JC, Fry B, Maller J, Daly MJ. Haploview: analysis and visualization of LD and haplotype maps. Bioinformatics. 2005 1 15;21(2):263–5. . 15297300 10.1093/bioinformatics/bth457

[32] Chen QH, Deng W, Li XW, Liu XF, Wang JM, Wang LF, et al Novel CDH1 germline mutations identified in Chinese gastric cancer patients. World journal of gastroenterology: WJG. 2013 2 14;19(6):909–16. Pubmed Central PMCID: 3574889. 10.3748/wjg.v19.i6.909 23431106 PMC3574889

[33] Black MD, Kaneshiro R, Lai JI, Shimizu DM. Hereditary diffuse gastric cancer associated with E-cadherin germline mutation: a case report. Hawai'i journal of medicine & public health: a journal of Asia Pacific Medicine & Public Health. 2014 7;73(7):204–7. . Pubmed Central PMCID: 4100282. 25089230 PMC4100282

[34] Cui Y, Xue H, Lin B, Ni P, Fang JY. A meta-analysis of CDH1 C-160A genetic polymorphism and gastric cancer risk. DNA and cell biology. 2011 11;30(11):937–45. 10.1089/dna.2011.1257 21612411

[35] Zhang XF, Wang YM, Ge H, Cao YY, Chen ZF, Wen DG, et al Association of CDH1 single nucleotide polymorphisms with susceptibility to esophageal squamous cell carcinomas and gastric cardia carcinomas. Diseases of the esophagus: official journal of the International Society for Diseases of the Esophagus / ISDE. 2008;21(1):21–9. . 18197935 10.1111/j.1442-2050.2007.00724.x

[36] Zhan Z, Wu J, Zhang JF, Yang YP, Tong S, Zhang CB, et al CDH1 gene polymorphisms, plasma CDH1 levels and risk of gastric cancer in a Chinese population. Molecular biology reports. 2012 8;39(8):8107–13. 10.1007/s11033-012-1658-0 22535324

[37] Lei H, Sjoberg-Margolin S, Salahshor S, Werelius B, Jandakova E, Hemminki K, et al CDH1 mutations are present in both ductal and lobular breast cancer, but promoter allelic variants show no detectable breast cancer risk. International journal of cancer Journal international du cancer. 2002 3 10;98(2):199–204. . 11857408 10.1002/ijc.10176

[38] Goode EL, Dunning AM, Kuschel B, Healey CS, Day NE, Ponder BA, et al Effect of germ-line genetic variation on breast cancer survival in a population-based study. Cancer research. 2002 6 1;62(11):3052–7. . 12036913

[39] International HapMap C. The International HapMap Project. Nature. 2003 12 18;426(6968):789–96. . 14685227 10.1038/nature02168

[40] Nicoloso MS, Sun H, Spizzo R, Kim H, Wickramasinghe P, Shimizu M, et al Single-nucleotide polymorphisms inside microRNA target sites influence tumor susceptibility. Cancer research. 2010 4 1;70(7):2789–98. Pubmed Central PMCID: 2853025. 10.1158/0008-5472.CAN-09-3541 20332227 PMC2853025

[41] Wang S, Tian Y, Wu D, Zhu H, Luo D, Gong W, et al Genetic variation of CTNNB1 gene is associated with susceptibility and prognosis of gastric cancer in a Chinese population. Mutagenesis. 2012 11;27(6):623–30. 10.1093/mutage/ges027 22848100

[42] Sauna ZE, Kimchi-Sarfaty C. Understanding the contribution of synonymous mutations to human disease. Nature reviews Genetics. 2011 10;12(10):683–91. 10.1038/nrg3051 21878961

[43] Mostowska A, Pawlik P, Sajdak S, Markowska J, Pawalowska M, Lianeri M, et al An analysis of polymorphisms within the Wnt signaling pathway in relation to ovarian cancer risk in a Polish population. Molecular diagnosis & therapy. 2014 2;18(1):85–91. . Pubmed Central PMCID: 3899496. 24078348 10.1007/s40291-013-0059-y PMC3899496

[44] Ting WC, Chen LM, Pao JB, Yang YP, You BJ, Chang TY, et al Common genetic variants in Wnt signaling pathway genes as potential prognostic biomarkers for colorectal cancer. PloS one. 2013;8(2):e56196 Pubmed Central PMCID: 3566082. 10.1371/journal.pone.0056196 23405266 PMC3566082

[45] Dapic V, Carvalho MA, Monteiro AN. Breast cancer susceptibility and the DNA damage response. Cancer control: journal of the Moffitt Cancer Center. 2005 4;12(2):127–36. . 15855896 10.1177/107327480501200210

