# Supplementary material for: Association of Genetic Polymorphisms in CDH1 and CTNNB1 with Breast Cancer Susceptibility and Patients' Prognosis among Chinese Han Women
Source: PLoS One. 2015 Aug 18;10(8):e0135865. doi: 10.1371/journal.pone.0135865 (PMC4540443; doi:10.1371/journal.pone.0135865)
Supplement: S1 Table — (DOC) [file pone.0135865.s001.doc]

**S1 Table. Characteristics of breast cancer cases and cancer-free controls.**

| Variable | Case, n=1160 | Control, n=1336 | *P* value |
| --- | --- | --- | --- |
| Age, years (mean ± SD) | 49.26±10.51 | 49.02±9.59 | 0.3973 |
| BMI (mean ± SD) | 24.58±3.22 | 24.54±3.50 | 0.6611 |
| Age at menarche, years (mean ± SD) | 14.54±1.82 | 15.07±1.90 | **0.0001** |
| Age at menopause, years (mean ± SD) | 49.15±4.28 | 49.19±3.83 | 0.8451 |
| Age of first birth, years (mean ± SD) | 26.12±2.86 | 25.42±2.77 | **<.0001** |
| Menopause status, n (%) |  |  | 0.6290 |
| Premenopause | 594 (51.21) | 685 (51.89) |  |
| Postmenopause | 566 (48.79) | 635 (48.11) |  |
| Number of childbirth, n (%) |  |  | 0.3621 |
| 0 | 25 (2.16) | 24 (1.80) |  |
| ≥1 | 1135 (97.84) | 1312 (98.20) |  |
| Family history of cancer in first-degree relatives, n (%) |  |  | **0.0064** |
| Yes | 249 (21.50) | 246 (18.41) |  |
| No | 909 (78.50) | 1090 (81.59) |  |
| Estrogen receptor (ER), n (%) |  |  |  |
| Positive | 621 (53.53) |  |  |
| Negative | 237 (20.43) |  |  |
| Missing data | 302 (26.04) |  |  |
| Progesterone receptor (PR), n (%) |  |  |  |
| Positive | 562 (48.45) |  |  |
| Negative | 292 (25.17) |  |  |
| Missing data | 306 (26.38) |  |  |
| human epidermal growth factor receptor 2 (Her2), n (%) |  |  |  |
| Normal | 621 (53.53) |  |  |
| Higher than normal | 234 (20.17) |  |  |
| Missing data | 305 (26.30) |  |  |
| The maximum diameter of the tumor, n (%) |  |  |  |
| ≤ 2 cm | 382 (32.93) |  |  |
| > 2 cm | 502 (43.28) |  |  |
| Missing data | 276 (23.79) |  |  |
| Clinical stage at diagnosis, n (%) |  |  |  |
| 0-I | 141 (12.16) |  |  |
| II-IV | 654 (56.38) |  |  |
| Missing data | 365 (31.46) |  |  |
| Lymph node metastasis, n (%) |  |  |  |
| Negative | 457 (39.40) |  |  |
| Positive | 325 (28.02) |  |  |
| Missing data | 378 (32.58) |  |  |
| Chemotherapy, n (%) |  |  |  |
| Yes | 822 (70.82) |  |  |
| No | 144 (12.41) |  |  |
| Missing data | 194 (16.77) |  |  |
| Endocrine therapy, n (%) |  |  |  |
| Yes | 233 (20.08) |  |  |
| No | 850 (73.28) |  |  |
| Missing data | 77 (6.64) |  |  |

Bold numbers indicate a statistical significance at 0.05 level.
