# Supplementary material for: Association of Genetic Polymorphisms in CDH1 and CTNNB1 with Breast Cancer Susceptibility and Patients' Prognosis among Chinese Han Women
Source: PLoS One. 2015 Aug 18;10(8):e0135865. doi: 10.1371/journal.pone.0135865 (PMC4540443; doi:10.1371/journal.pone.0135865)
Supplement: S2 Table — (DOC) [file pone.0135865.s002.doc]

***S2 Table. Hardy-Weinberg equilibrium analysis of the SNPs in CDH1 and CTNNB1.***

| Gene | SNP | *P* value | HWE |
| --- | --- | --- | --- |
| *CDH1* | rs7200690 | 0.2388 | Yes |
|  | rs12185157 | 0.2157 | Yes |
|  | rs7198799 | 0.4899 | Yes |
|  | rs17715799 | 0.1025 | Yes |
|  | rs10431923 | 0.4113 | Yes |
|  | rs7186053 | 0.5798 | Yes |
|  | rs6499199 | 0.5288 | Yes |
|  | rs4783689 | 0.1566 | Yes |
|  | rs13689 | 0.2696 | Yes |
| *CTNNB1* | rs4533622 | 0.1555 | Yes |
|  | rs4135385 | 0.5817 | Yes |
|  | rs2293303 | 0.7137 | Yes |
