# Supplementary material for: Association of Genetic Polymorphisms in CDH1 and CTNNB1 with Breast Cancer Susceptibility and Patients' Prognosis among Chinese Han Women
Source: PLoS One. 2015 Aug 18;10(8):e0135865. doi: 10.1371/journal.pone.0135865 (PMC4540443; doi:10.1371/journal.pone.0135865)
Supplement: S3 Table — (DOC) [file pone.0135865.s003.doc]

# S3 Table. Frequencies of diplotype of block1 in *CDH1* and its association with risk of breast cancer.

| Diplotype ^a^ | Cases (%) | Controls (%) | *P* value | OR (95% CI) | *P* value | aOR (95% CI) ^b^ | *P* value ^b^ |
| --- | --- | --- | --- | --- | --- | --- | --- |
| CAC-CAC | 313 (26.98) | 376 (28.14) | **0.0004** |  |  |  |  |
| CAC-CGC | 297 (25.600 | 349 (26.12) |  | 1.02 (0.82-1.27) | 0.8411 | 1.03 (0.83-1.28) | 0.7610 |
| CAC-TGT | 146 (12.59) | 174 (13.02) |  | 1.01 (0.77-1.32) | 0.9534 | 1.01 (0.77-1.32) | 0.9600 |
| CGC-CGC | 102 (8.79) | 106 (7.93) |  | 1.16 (0.85-1.58) | 0.3603 | 1.17 (0.86-1.59) | 0.3320 |
| CAC-TGC | 70 (6.03) | 103 (7.71) |  | 0.82 (0.58-1.15) | 0.2404 | 0.83 (0.59-1.16) | 0.2775 |
| CGC-TGT | 62 (5.34) | 102 (7.63) |  | 0.73 (0.52-1.04) | 0.0778 | 0.74 (0.52-1.05) | 0.0884 |
| CGC-TGC | 47 (4.05) | 36 (2.69) |  | 1.57 (0.99-2.48) | **0.0548** | 1.59 (1.01-2.52) | **0.0472** |
| TGT-TGT | 34 (2.93) | 27 (2.02) |  | 1.51 (0.89-2.56) | 0.1237 | 1.52 (0.90-2.58) | 0.1189 |
| TGT-TGC | 15 (1.29) | 23 (1.72) |  | 0.78 (0.40-1.53) | 0.4736 | 0.79 (0.41-1.55) | 0.4996 |
| Others | 74 (6.38) | 40 (2.98) |  | 2.22 (1.47-3.36) | 0.0001 | 2.26(1.495-3.42) | 0.0001 |

^a^ Diplotypes in *CDH1 Block 1* [ rs7200690(C>T) + rs12185157(A>G)+rs7198799(C>T)].

^b^ Adjusted for age at menarche, age of first birth and family history of cancer in first-degree relatives.

Bold numbers indicate a statistical significance at 0.05 level.
