# Supplementary material for: Association of Genetic Polymorphisms in CDH1 and CTNNB1 with Breast Cancer Susceptibility and Patients' Prognosis among Chinese Han Women
Source: PLoS One. 2015 Aug 18;10(8):e0135865. doi: 10.1371/journal.pone.0135865 (PMC4540443; doi:10.1371/journal.pone.0135865)
Supplement: S4 Table — (DOC) [file pone.0135865.s004.doc]

**S4 Table. Univariate and Multivariate Cox proportional hazard analysis of the SNPs and haplotypes in *CDH1* and *CTNNB1* in relation to event-free survival of breast cancer patients.**

| Parameter | | No. | No_event_ (%) | | Log-rank *P* value | | HR (95%CI) | | *P* value | | aHR (95%CI)* | *P* value* |
| --- | --- | --- | --- | --- | --- | --- | --- | --- | --- | --- | --- | --- |
| rs7200690 | |  |  | |  | | |  |  |  | |  |
| CC | | 704 | 98 (14) | | 0.1388 | | |  |  |  | |  |
| CT | | 311 | 49 (16) | |  | | | 1.20 (0.85-1.69) | 0.3091 | 1.39 (0.78-2.46) | | 0.2678 |
| TT | | 76 | 16 (21) | |  | | | 1.66 (0.98-2.81) | 0.0620 | 2.41 (0.997-5.82) | | 0.0507 |
| CT/TT vs. CC (dominant model) | |  |  | |  | | | 1.29 (0.94-1.76) | 0.1183 | 1.55 (0.91-2.62) | | 0.1067 |
| TT vs. CT/CC (recessive model) | |  |  | |  | | | 1.57 (0.94-2.63) | 0.0886 | 2.23 (0.95-5.27) | | 0.0668 |
| rs12185157 | |  |  | |  | | |  |  |  | |  |
| AA | | 317 | 53 (17) | | 0.3398 | | |  |  |  | |  |
| AG | | 506 | 68 (13) | |  | | | 0.82 (0.56-1.21) | 0.3155 | 1.13 (0.58-2.22) | | 0.7132 |
| GG | | 268 | 42 (16) | |  | | | 1.06 (0.71-1.59) | 0.7808 | 1.14 (0.56-2.33) | | 0.7147 |
| AG/GGvs. AA (dominant model) | |  |  | |  | | | 0.91(0.64-1.30) | 0.6048 | 1.14 (0.61-2.12) | | 0.6846 |
| GG vs. AG/AA (recessive model) | |  |  | |  | | | 1.20 (0.87-1.67) | 0.2734 | 1.05 (0.60-1.84) | | 0.8565 |
| rs7198799 | |  |  | |  | | |  |  |  | |  |
| CC | | 807 | 125 (15) | | 0.8672 | | |  |  |  | |  |
| CT | | 240 | 31 (13) | |  | | | 0.90 (0.61-1.34) | 0.6117 | 0.98 (0.50-1.90) | | 0.9505 |
| TT | | 44 | 7 (16) | |  | | | 1.04 (0.49-2.24) | 0.9116 | 2.17 (0.52-9.15) | | 0.2908 |
| CT/TT vs. CC (dominant model) | |  |  | |  | | | 0.93 (0.64-1.33) | 0.6790 | 1.07 (0.58-1.99) | | 0.8302 |
| TTvs. CT/CC` (recessive model) | |  |  | |  | | | 1.07 (0.50-2.28) | 0.8678 | 2.18 (0.52-9.14) | | 0.2865 |
| rs17715799 | |  |  | |  | | |  |  |  | |  |
| AA | | 737 | 112 (15) | | 0.3293 | | |  |  |  | |  |
| AT | | 283 | 44 (16) | |  | | | 1.11 (0.78-1.58) | 0.5513 | 0.92 (0.44-1.55) | | 0.5450 |
| TT | | 71 | 7 (10) | |  | | | 0.61 (0.29-1.31) | 0.2063 | 1.17 (0.36-3.81) | | 0.7962 |
| AT/TT vs. AA (dominant model) | |  |  | |  | | | 0.998 (0.72-1.39) | 0.9903 | 0.87 (0.49-1.56) | | 0.6470 |
| TT vs. AT/AA (recessive model) | |  |  | |  | | | 0.59 (0.28-1.27) | 0.1780 | 1.23 (0.38-3.96) | | 0.7350 |
| rs10431923 | |  |  | |  | | |  |  |  | |  |
| TT | | 362 | 60 (17) | | 0.6430 | | |  |  |  | |  |
| GT | | 508 | 74 (15) | |  | | | 1.07 (0.69-1.64) | 0.7720 | 1.29 (0.62-2.69) | | 0.4973 |
| GG | | 221 | 29 (13) | |  | | | 1.21 (0.78-1.89) | 0.3987 | 1.28 (0.61-2.71) | | 0.5164 |
| GT/GG vs. TT (dominant model) | |  |  | |  | | | 1.13 (0.75-1.68) | 0.5639 | 1.29 (0.65-2.55) | | 0.4712 |
| GG vs. GT/TT (recessive model) | |  |  | |  | | | 1.16 (0.84-1.59) | 0.3705 | 1.08 (0.63-1.84) | | 0.7889 |
| rs7186053 | |  |  | |  | | |  |  |  | |  |
| GG | | 569 | 92(16) | | 0.5823 | | |  |  |  | |  |
| AG | | 399 | 53(13) | |  | | | 0.84 (0.60-1.18) | 0.3068 | 0.80 (0.44-1.46) | | 0.4741 |
| AA | | 123 | 18(15) | |  | | | 0.98 (0.59-1.63) | 0.9421 | 0.64 (0.25-1.63) | | 0.3468 |
| AG/AAvs. GG (dominant model) | |  |  | |  | | | 0.87 (0.64-1.19) | 0.3804 | 0.75 (0.44-1.30) | | 0.3100 |
| AA vs. AG/GG (recessive model) | |  |  | |  | | | 1.05 (0.64-1.72) | 0.8418 | 0.68 (0.27-1.72) | | 0.4157 |
| rs6499199 | |  |  | |  | | |  |  |  | |  |
| CC | | 838 | 122(15) | | 0.6995 | | |  |  |  | |  |
| CT | | 223 | 37(17) | |  | | | 1.17 (0.81-1.69) | 0.4029 | 0.95 (0.49-1.84) | | 0.8714 |
| TT | | 30 | 4(13) | |  | | | 0.98 (0.36-2.65) | 0.9650 | 0.00 | | 0.9841 |
| CT/TT vs. CC (dominant model) | |  |  | |  | | | 1.15 (0.81-1.64) | 0.4446 | 0.85 (0.44-1.66) | | 0.6413 |
| TT vs. CT/CC (recessive model) | |  |  | |  | | | 0.95 (0.35-2.55) | 0.9126 | 0.00 | | 0.9841 |
| rs4783689 | |  |  | |  | | |  |  |  | |  |
| CC | | 541 | 100(18) | | **0.0152** | | |  |  |  | |  |
| CT | | 433 | 49(11) | |  | | | 0.62 (0.44-0.87) | **0.0054** | 0.75 (0.42-1.33) | | 0.3264 |
| TT | | 117 | 14(12) | |  | | | 0.70 (0.40-1.23) | 0.2130 | 0.75 (0.29-1.92) | | 0.5452 |
| CT/TT vs. CC (dominant model) | |  |  | |  | | | 0.63 (0.46-0.87) | **0.0045** | 0.75 (0.44-1.27) | | 0.2863 |
| TT vs. CT/CC (recessive model) | |  |  | |  | | | 0.85 (0.49-1.46) | 0.5490 | 0.83 (0.33-2.09) | | 0.6911 |
| rs13689 | |  |  | |  | | |  |  |  | |  |
| TT | | 720 | 109(15) | | **0.0311** | | |  |  |  | |  |
| CT | | 309 | 39(13) | |  | | | 0.78 (0.54-1.13) | 0.1932 | 0.55 (0.28-1.09) | | 0.0846 |
| CC | | 62 | 15(24) | |  | | | 1.73 (1.01-2.96) | **0.0474** | 0.57 (0.14-2.35) | | 0.4339 |
| CT/CC vs. TT (dominant model) | |  |  | |  | | | 0.93 (0.67-1.29) | 0.6463 | 0.55 (0.29-1.04) | | 0.0664 |
| CC vs. CT/TT (recessive model) | |  |  | |  | | | 1.85 (1.09-3.15) | **0.0233** | 0.62 (0.15-2.54) | | 0.5043 |
| rs4533622 | |  |  | |  | | |  |  |  | |  |
| CC | | 687 | 99 (14) | | 0.5302 | | |  |  |  | |  |
| AC | | 338 | 56 (17) | |  | | | 1.14 (0.82-1.59) | 0.4296 | 1.12 (0.75-1.68) | | 0.5882 |
| AA | | 66 | 8 (12) | |  | | | 0.84 (0.41-1.72) | 0.6369 | 1.26 (0.61-2.64) | | 0.5346 |
| AC/AA vs. CC (dominant model) | |  |  | |  | | | 1.09 (0.80-1.50) | 0.5818 | 1.14 (0.78-1.65) | | 0.4941 |
| AA vs. AC/CC (recessive model) | |  |  | |  | | | 0.80 (0.39-1.63) | 0.5448 | 1.22 (0.59-2.50) | | 0.5963 |
| rs4135385 | |  |  | |  | | |  |  |  | |  |
| GG | | 282 | 38 (13) | | 0.7248 | | |  |  |  | |  |
| AG | | 562 | 87 (15) | |  | | | 1.18 (0.81-1.79) | 0.3942 | 1.10 (0.70-1.74) | | 0.6723 |
| AA | | 247 | 38 (15) | |  | | | 1.21 (0.77-1.91) | 0.4051 | 0.96 (0.55-1.65) | | 0.8697 |
| AG/AAvs. GG (dominant model) | |  |  | |  | | | 1.19 (0.83-1.72) | 0.3504 | 1.06 (0.68-1.63) | | 0.8076 |
| AA vs. AG/GG (recessive model) | |  |  | |  | | | 1.08 (0.75-1.56) | 0.6741 | 0.89 (0.57-1.40) | | 0.6204 |
| rs2293303 | |  |  | |  | | |  |  |  | |  |
| CC | | 835 | 123 (15) | | 0.9266 | | |  |  |  | |  |
| TC | | 226 | 35 (15) | |  | | | 1.04 (0.72-1.52) | 0.8332 | 0.99 (0.62-1.58) | | 0.9564 |
| TT | | 30 | 5 (17) | |  | | | 1.16 (0.48-2.85) | 0.7405 | 1.87 (0.75-4.64) | | 0.1771 |
| TC/TT vs. CC (dominant model) | |  |  | |  | | | 1.06 (0.74-1.51) | 0.7687 | 1.08 (0.70-1.67) | | 0.7253 |
| TT vs. TC/CC (recessive model) | |  |  | |  | | | 1.15 (0.47-2.81) | 0.7541 | 1.88 (0.76-4.63) | | 0.1728 |
| *CDH1* Block1 |  | | |  | |  | |  |  |  | |  |
| CAC | | 1097 | 166(15) | | 0.0911 | | |  |  |  | |  |
| CGC | | 585 | 75(13) | |  | | | 0.84 (0.64-1.10) | 0.2086 | 0.81 (0.51-1.29) | | 0.3775 |
| TGT | | 278 | 41(15) | |  | | | 1.02 (0.72-1.43) | 0.9188 | 1.23 (0.70-2.17) | | 0.4788 |
| TGC | | 151 | 33(22) | |  | | | 1.49 (1.02-2.18) | **0.0380** | 1.36 (0.71-2.59) | | 0.3558 |
| Others | | 71 | 11(15) | |  | | | 1.23 (0.67-2.26) | 0.5140 | 1.81 (0.65-4.99) | | 0.2554 |

*** Adjusted for ER status, PR status, Her2 status, tumor size, clinical stage, lymphnode metastasis, chemotherapy and endocrine therapy.

Bold numbers indicate a statistical significance at 0.05 level.
